# Too young for medication? prescriber perspectives on how age shapes medication for opioid use disorder prescribing in transition-age adults

**DOI:** 10.1186/s12954-026-01467-1

**Published:** 2026-05-22

**Authors:** Diego Renteria, Adetayo Fawole, Jason Alexandre, Haley Allen, Joshua Aleksanyan, Sugy Choi, Edward Liebmann, Sueun Hong, Ashly E. Jordan, Megan O’ Grady, Vanessa Drury, Sam Kawola, Jamie del Rosario, Pat Lincourt, Charles J. Neighbors

**Affiliations:** 1 NYU Grossman School of Medicine, New York, NY USA; 2 Department of Population Health, NYU Grossman School of Medicine, 180 Madison Ave, Suite 350, New York, NY 10016 USA; 3 New York State Psychiatric Institute, New York, NY USA; 4 Office of Addiction Services and Supports, New York State, New York, NY USA; 5 Department of Public Health Sciences, UConn School of Medicine, Farmington, CT USA

## Abstract

**Background:**

Transition-age (TA) adults are less likely than adults aged 26 and older to receive medications for opioid use disorder (MOUD), and among those treated, disproportionately receive naltrexone over more effective agonist MOUD (buprenorphine and methadone). Given that prescribers ultimately determine medication selection, understanding how age influences their clinical decisions is essential to addressing these disparities. This study examines how prescribers incorporate age into MOUD prescribing decisions for this developmentally vulnerable population.

**Methods:**

Using 2022 New York State Medicaid claims, we identified MOUD prescribers with substantial experience (treating n ≥ 5 TA adults) and conducted semi-structured interviews with 18 outpatient prescribers (MD/DOs, NPs, PAs) from diverse geographic and clinical settings. We applied an inductive thematic analysis approach to explore how prescribers incorporated age and age-related factors into their clinical decision-making around MOUD.

**Results:**

Most prescribers did not endorse chronological age as a factor influencing their prescribing patterns, but rather described tailoring treatment based on age-related factors more commonly seen in TA adults. These included developmental characteristics (perceived lack of commitment, peer influence), socioeconomic barriers (e.g., unreliable transportation leading to missed appointments), family influence, and concerns about MOUD dependence. In response to these age-related factors, prescribers implemented harm-reduction principles by adjusting MOUD type prescribing to accommodate TA adults' life-stage realities. In contrast, a small subset of prescribers explicitly cited chronological age as directly influencing MOUD prescribing, expressing hesitations about initiating buprenorphine or referring TA adults for methadone due to long-term dependence concerns.

**Conclusion:**

These findings suggest two key mechanisms through which age may influence MOUD prescribing disparities among TA adults: (1) prescribers' responses to age-related life-stage challenges (e.g., developmental and social factors, structural barriers) and (2) explicit prescriber age-based hesitancy about initiating long-term agonist maintenance in younger patients. The first mechanism appeared more influential than explicit hesitancy. These mechanisms may help explain documented disparities in MOUD type prescribed. Addressing these disparities may require multi-level interventions: prescriber education affirming agonist MOUD effectiveness for TA adults, efforts to reduce MOUD misconceptions among patients and families, peer-based approaches that leverage social influence positively, and flexible harm-reduction-oriented clinic policies that accommodate structural barriers while supporting access to evidence-based MOUD.

## Introduction

Healthcare providers are key players in determining access to medications for opioid use disorder (MOUD), which consist of methadone, buprenorphine, and naltrexone. Their MOUD prescribing decisions are shaped by multiple factors, including their medical training, how they interpret treatment guidelines, and their own views about addiction treatment [1–3]. Although clinical evidence has overwhelmingly shown MOUD to be the most effective treatments for opioid use disorder (OUD), significant variability in MOUD prescribing occurs between patient populations [4–6]. Understanding how providers make decisions, and what factors they consider when deciding what type of MOUD to prescribe, is essential to addressing disparities in treatment access, retention, and outcomes.

One population where MOUD prescribing disparities are particularly apparent is in transition-age (TA) adults (ages 18—25), a group also referred to in the literature as emerging adults and young adults [7]. This age range is in a distinct clinical and developmental period, differing from adolescents in pediatric care and older adults with more stability, in which early substance use and healthcare transitions increase overdose risk and shape MOUD receipt [8, 9]. TA adults have been disproportionately affected by the opioid epidemic, as peak incidence of OUD occurs during this developmental period and overall OUD prevalence is greater than that of adults aged 26 years and older. [10–13]. TA adults also have an elevated risk of overdose compared to older adults, with one study following opioid-naïve individuals receiving a first opioid prescription, adults aged 18–25 had approximately an 80% higher risk of fatal or nonfatal opioid overdose compared with adults aged 25–44 [14].

Given the elevated incidence of OUD and overdose risk among TA adults, and evidence that MOUD can significantly reduce both overdose risk by up to 86% and all-cause mortality by over 50%, TA adults nonetheless receive MOUD at significantly lower rates than adults older than 26 years [5, 15–19]. To quantify this disparity, one study using national surveillance data shows that among adults who needed and received OUD treatment, only 19.9% of TA adults received medications for OUD compared with 68.4% of adults aged 35–49, showing a greater than threefold difference in MOUD receipt by age [20]. Importantly, among TA adults who do receive MOUD, disparities persist between TA adults and older adults in the type of MOUD received. In one study of patients treated with MOUD following nonfatal overdose, those aged 18–21 and 22–25 were respectively 65% and 41% more likely to receive naltrexone compared with adults aged 26–45. [21]. This disparity is concerning as naltrexone has not been associated with reduced opioid-related or all-cause mortality, while opioid agonist MOUD (buprenorphine and methadone) have been shown to be superior for overdose prevention and reducing all-cause mortality when compared to Naltrexone [19, 22, 23]. Given that MOUD is an essential harm reduction intervention for OUD, age-related disparities in the type of MOUD prescribed effectively reduce access to the most effective harm reduction MOUD treatments for TA adults [24]. Taken together, TA adults are simultaneously at heightened risk for overdose, less likely to receive MOUD, and more likely to receive the MOUD with the weakest evidence for mortality reduction. This misalignment between clinical risk and treatment effectiveness may be contributing to preventable overdose morbidity and mortality, underscoring the urgent need for focused research addressing MOUD prescribing disparities in TA adults.

While disparities in overall MOUD receipt have been studied extensively, an important and underexplored question is why, even when TA adults do receive MOUD, differences persist in the type of medication prescribed. Because these differences arise only after care has been initiated, they necessarily reflect medication-specific clinical decisions made by prescribers, who represent the final and unavoidable decision point in MOUD selection. Understanding how patient characteristics, and particularly age, shape these prescribing decisions is therefore essential to understanding and ultimately addressing age-related disparities in the type of MOUD received by TA adults. Without this insight, harm reduction efforts risk overlooking the clinical judgments and other factors directly determining whether TA adults receive the most effective MOUD types, restricting our ability to design effective harm reduction interventions that improve OUD treatment outcomes for TA adults.

This study examines how the age of TA adults shapes clinical decision-making among providers who prescribe MOUD to this population, including whether age influences MOUD type selection and whether it contributes to prescribing hesitancy. Through qualitative interviews with prescribers who treat TA adults, we identify age-related perceptions, biases, and clinical considerations that may be contributing to these MOUD prescribing disparities. Ultimately, these findings can help inform prescriber and system-level interventions to improve equitable receipt of effective MOUD for this underserved population.

## Methods

### Study sample selection

This study analyzed New York State Medicaid pharmacy claims from 2022 to identify prescribers who prescribed any formulation (i.e., oral, intramuscular) buprenorphine or naltrexone to TA adults who were defined as individuals aged 18 to 25 as of January 1, 2022. Methadone was not a variable used as it was not recorded and captured in Medicaid pharmacy claims. This allowed us to focus on MOUD treatment outside of opioid treatment programs (OTPs), as most MOUD prescribing occurs outside of OTPs [25]. The prescribers in our sample cannot prescribe methadone; however, they serve as an important referral pathway for methadone treatment. Thus, their perspectives on methadone for TA adults is relevant and necessary to understanding MOUD disparities for this age group. To identify experienced MOUD prescribers, we defined these prescribers with substantial experience (Physicians (MDs and DOs), Nurse Practitioners (NPs), and Physician Assistants (PAs)) as those who prescribed buprenorphine or naltrexone to at least five TA adults (n ≥ 5) in 2022, a threshold that was chosen to enhance the sensitivity of the study. While Medicaid data captures only a portion of a prescriber’s overall practice, this threshold served as a proxy for recent and significant experience prescribing to this age group. It attempts to balance external validity and depth by including prescriber types with a broader range of prescribing experience while excluding prescribers with incidental or minimal experience. Applying the n ≥ 5 criterion, we identified a final subset of 104 prescribers who comprised our final recruitment sample, all of whom were contacted for study participation. Additional sample selection methodological details are available in Appendix A.

### Study design

This qualitative study used Criterion-i purposeful sampling to identify prescribers with substantial experience treating TA adults [26]. Of the identified prescribers through Medicaid data, the only exclusion criterion was retirement from clinical work. This study is part of a larger study examining substance use disorder (SUD) treatment for TA adults in New York State and was approved by the Institutional Review Board at the New York University Grossman School of Medicine (#i2200914) [27].

### Data collection

For this study, we conducted interviews with 18 prescribers representing a range of counties and SUD treatment settings across New York State. Of the identified prescribers using Medicaid administrative data, contact information was obtained via an internet search, as no prior directories or clinic connections were previously established. All prescribers were contacted through at least one method, either through phone, email, letters, or rarely, clinic leadership. Recruitment began in February 2024 and ended in May 2024 after reaching saturation. Data saturation was determined through interview team debriefings where the research team noted that little new information related to our research questions was being elicited [28]. Of the 104 prescribers we attempted to contact, 18 participated (17.3%), 82 never responded, and 4 prescribers declined participation. Of these 4 prescribers who declined, 1 cited time constraints, another cited disinterest, and 2 did not provide a reason for declining participation.

A semi-structured interview guide was developed to align with study objectives, informed by a phased approach to interview guide development, and drawing from prior interview guides in MOUD and SUD treatment to guide structure and content [29–31]. The guide underwent multiple revisions based on feedback and pilot testing from MOUD-prescribing clinicians and researchers. The interview guide explored treatment approaches, age, and patient factors influencing MOUD clinical decision-making, and challenges in prescribing MOUD to Medicaid and non-Medicaid insured TA adults in their routine clinical practice. Each prescriber participated in a single 30-45 min, one-on-one interview via Zoom with author AF (male, qualitative expert) or author DR (male, trained medical student). Both interviewers were experienced in qualitative methods, with their previous MOUD research and clinical experiences likely influencing their interview approach and interpretation of prescriber perspectives. Field notes were taken during and after finishing interviews. Verbal informed consent was obtained prior to audio recording. All interviews were professionally transcribed, anonymized, and uploaded to Dedoose for coding. Dedoose is a secure, HIPAA-compliant platform for qualitative data analysis. Participants received a $100 e-gift card as compensation for participation.

### Data analysis

We conducted a thematic analysis following the guidelines described by Nowell et al. (2017) to identify key themes emerging from prescriber interviews [32]. The research team (author AF, author DR, author HA, author SC) collaborated to develop an initial coding framework. Using an inductive approach, two interviewers (author AF, author DR) independently reviewed the first transcript, noting preliminary themes and concepts. The team then met to discuss and refine these themes, repeating this process with three additional transcripts to build a robust codebook. Once finalized, two researchers independently applied the codebook to the first transcript to ensure clarity and consistency, reconciling discrepancies before proceeding. Coding was conducted by one qualitative expert (author AF) and two trained medical students (authors DR and HA). Following common best practices, early collaborative double-coding was conducted, and exactly 50% of transcripts were double-coded and reconciled to support reliability [33]. Final themes were developed through a comprehensive review of coded data, with team discussions ensuring they accurately reflected participants' views and experiences.

## Results

As shown in Table 1, 18 prescribers participated in the study. In the sample, 3 participants identified as male, and the average age across all participants was 47.88 years. Most participants (78%) identified as non-Hispanic white. The group included 9 nurse practitioners (NPs), 3 physician assistants (PAs), and 6 physicians (MDs or DOs). A total of 6 participants (33.3%) practiced in rural counties as defined by New York State (population < 200,000), with the other 12 practicing in suburban and urban counties [34]. The two main themes identified were: (1) The role of a patient’s chronological age on MOUD prescribing decisions and (2) Prescriber perceptions of patient preferences and attitudes towards MOUD during TA adulthood.

**Table 1:** Participant Characteristics

Characteristic	%	n
Gender		
Male	16.7	3
Female	83.3	15
Race/Ethnicity		
Non-Hispanic White	78	14
Non-Hispanic Black	11.1	2
Hispanic	11.1	2
Prescriber Type		
Nurse Practitioners (NPs)	50.0	9
Physician Assistants (PAs)	16.7	3
Medical Doctors (MD/DOs)	33.3	6
Practice Setting		
Rural	33.3	6
Average Age (Mean, SD)		47.88 (8.2)
TA adults prescribed MOUD per prescriber in NYS Medicaid in 2022, mean (median, range)	**7**.2 (6, 5—12)	7.2 (6, 5—12)

### The role of a patient’s chronological age on MOUD prescribing decisions

Prescribers shared various perspectives on the role of a patient’s chronological age as a factor influencing MOUD prescribing decisions.

#### Chronological age as a standalone factor in MOUD prescribing

When specifically prompted by interviewers ('Does the age of the patient you are prescribing for factor into that decision?'), most prescribers interviewed (16 of 18, 89%) did not list patient’s chronological age as a direct consideration in determining appropriate MOUD. Many prescribers stated that they approached TA adults similarly to adults older than 25 in their prescribing practices. These prescribers described guiding decisions primarily by clinical criteria, patient willingness, and the treatment options available. One prescriber explained, “…their age doesn’t really come into it. Like, if they’re 18 to 25 years old, their age doesn’t come into play for me of what [MOUD] is better for them.” (MD 10).

In contrast, a minority of prescribers interviewed (2 of 18, 11%) explicitly described personally using chronological age as a factor when making MOUD prescribing decisions. One prescriber shared their approach working with TA adults, stating: “But yeah, there are some that I’m like, ‘Hey, you know, you’re young, I don't want to put you on this, or you’re gonna be on it for years. Let’s at least try this first’…” (NP 01). Another prescriber stated that when triaging patients for MOUD, “…some of the ones that are approaching the age 25, or whatever, might be candidates, um, for the Methadone” (NP 02), implying that age itself is a factor determining which MOUD is most appropriate. Although not describing their own prescribing decisions, one prescriber described perceived hesitancy among peer clinicians to prescribe buprenorphine to younger patients or to refer them for methadone treatment. This hesitation was perceived to be linked to concerns about the long-term commitment and eventual challenges of tapering off. As this prescriber noted, “…I do know that some people feel like hesitant. Like, they might feel – the clinician might feel hesitant to, like, prescribe something that’s, like, gonna require tapering off later or something like that…” (NP 12). Although clinical reservations persist regarding prescribing certain MOUD to TA adults, some prescribers noted a shift toward greater acceptance of methadone. One prescriber shared that, “…I remember back in 2018, we would be like, ‘Wow… 22. That’s really young for methadone’…but now…it’s kind of whatever people feel is going to be – work for them” (NP 03).

#### Age-related factors that influence prescribing

*Life-Stage Factors* In contrast to instances where prescribers treated younger chronological age as an independent factor in MOUD prescribing decisions, most prescribing considerations described for TA adults reflected age-related developmental, social, and structural factors rather than chronological age itself. Multiple prescribers mentioned that they particularly favored flexible treatment options for youth because, “…you don’t have to go to a Methadone clinic every day…Buprenorphine is much, much easier…especially in that age range” (DO 15). Prescribers factored in how TA adults may be busier with “work” or “classes” and consequently recommended MOUD options to accommodate their life situation. Patients’ developmental characteristics were also described as factors that influenced the type of MOUD prescribed. One example of this affecting medication choice was when prescribers perceived that younger patients had difficulties adhering to daily medication regimens, making them better candidates for long-acting injectables (LAIs). “I think it’s not so much age but like, maturity, right?…some younger patients who really struggle to make the visits…who just have a hard time…being in treatment…so, for those patients…we will lean a little bit more towards like an injectable medication…” one prescriber explained (MD 17). Related to their early life stage, some prescribers found that TA adults appeared to be unsure about their substance use goals, with one prescriber sharing that, “…there’s an overarching theme of like they’re [TA adults] not ready. They’re just not ready to stop” (NP 14) and another sharing that, “…the younger population is still…into glorifying [substance use]” (NP 02).

*Socioeconomic Factors* Prescribers described adjusting treatment approaches in response to the financial instability commonly seen in TA adults. These age-related socioeconomic barriers were perceived by prescribers as leading to inconsistent medication use and “…a much higher don’t show rate to appointments” (NP 12), prompting some prescribers to alter medication regimens or, in some cases, consider treatment discontinuation. One prescriber noted that some TA adults, “…either they don’t have a car, or …regular transportation, or, um – and so they miss appointments and will sometimes get discharged from other practices…” (NP 06) with another prescriber sharing that, “If I see that they’re not following up and that I have to chase after them…if I’m doing more than you, then this [MOUD treatment] ain’t for you” (PA 11). Prescribers also adapted their prescribing strategies to accommodate the housing instability that frequently characterizes early adulthood, such as living in shared or unsafe environments that increase the risk of loss or theft of medication. In response, some prescribers reduced the quantity of take-home medications or switched to LAIs, reflecting flexible prescribing tailored to TA adults’ living situations.

*Family Influence on Treatment Decisions* The family living arrangements of TA adults further influenced MOUD decisions, particularly when patients resided with family members who did not support treatment. When prescribers reported this familial pressure, it was often against buprenorphine in particular, with patients reporting that their families viewed its agonist properties as “treating one drug with another” and feared dependence (MD 10). Prescribers perceived that this familial pressure could lead TA adults to request medications or tapering plans that did not fully reflect their own treatment desires. Some prescribers accelerated tapering or withheld certain medications at the request of patients who were under significant familial pressure, underscoring how family dynamics uniquely shape care decisions for this age group. As one prescriber explained, “Sometimes they have family members who say, ‘You need to be off [MOUD] by this time or I’m leaving you,’ so we’ll end up expediting the taper” (NP 01). While family influence was described as a barrier by some, many prescribers also recognized that strong familial support could enhance engagement and adherence to MOUD. One prescriber emphasized this dual role, stating, “I think…there really are amazingly supportive families…I think it does help with engagement…” (NP 06).

### Prescriber perceptions of patient preferences and attitudes towards MOUD during TA adulthood

Prescribers’ perceptions of patient preferences significantly influenced the MOUD clinical decision-making processes when working with TA adults. Many prescribers perceived that TA adults often wanted to avoid buprenorphine and methadone due to concerns of "dependence." As one prescriber noted, “…they don’t want to be dependent on it, so, they really want to be out of the opioid” (MD 18). Some prescribers also perceived that TA adults hesitated to start MOUD due to fears of lifelong medication use, with one prescriber sharing, “they don’t wanna be on medications for the rest of their life…” (PA 07). These perceived TA adult concerns about dependency and long-term treatment influenced how prescribers discussed MOUD options with patients, with some indicating they prescribed naltrexone over buprenorphine referrals to OTP for methadone when patients were especially resistant as evidenced by the following quote, “…some patients would tell me, ‘I don’t want Methadone. I don’t want Suboxone. I want Vivitrol because it’s not a opioid.’ And so, um, if they were requesting it by name, um, I would totally give it” (DO 15).

*Perception and Misinformation*: Patient misconceptions surrounding MOUD were frequently cited as a factor influencing prescribing choices, as some providers perceived TA adults as being more susceptible to negative views and peer influence. One provider shared that, “Like all – there’s a lot of resistance. [Laughs] Like, I would say, uh, more so than kind of other age groups…Um, just – and a lot of it is stigma and sort of what – what they are, they’re concerned about what other people might – might think of them” (MD 17). Another provider explained, “The peer pressure um, they haven’t formed as much of a self-identity um, as older patients, so I think [TA adults] are…more easily swayed…” (MD 13).

Although some prescribers perceived a higher incidence of negative views of MOUD among TA adults, others noted that this group appeared more open to using MOUD than adults older than 26 in certain cases, possibly because of changing cultural norms and increased access to information about MOUD. One prescriber shared, “I feel like the younger generation is a little bit more open to it because…the medications nowadays are a little bit more out there and available for the younger generation” (NP 01). Overall, prescribers described TA adults as both more influenced by peers and stigma, while also at times being more open to MOUD.

## Discussion

### Chronological age and explicit prescriber hesitancy in MOUD selection

A central question guiding this study was whether, and how, age influences MOUD prescribing practices when treating TA adults. We found that most prescribers in our sample did not endorse chronological age alone as a major factor influencing MOUD prescribing, which is itself a significant finding, as it suggests this group of prescribers may be moving away from traditional age-based biases that have historically limited TA adults’ access to buprenorphine and methadone therapies [35, 36]. However, this finding should be interpreted carefully as clinicians often minimize or under-report their own biases [37, 38].

Although only 2 of 18 prescribers (11%) explicitly cited age as influencing their prescribing decisions, even this limited presence of explicit bias may signal broader unspoken hesitancy that influences treatment decisions for TA adults. These prescribers expressed hesitancy about prescribing buprenorphine or referring younger patients to OTPs for methadone. Though providers were often motivated by concerns about preventing long-term dependence on agonist MOUD, these concerns were likely shaped by MOUD-related stigma toward maintenance treatment [39, 40]. Prescribers who share these views may, in turn, be more likely to favor naltrexone over agonist MOUD for TA adults. This may help explain the disproportionately higher use of naltrexone in this population, despite evidence demonstrating greater effectiveness of buprenorphine and methadone for TA adults [21, 22]. In this way, withholding agonist MOUD from TA adults reflects a form of institutionalized paternalism that conflicts with harm reduction principles and ultimately places TA adults at increased risk of morbidity and mortality [19, 41]. These findings highlight the dual reality that while many prescribers and addiction treatment institutions are reducing age-based restrictions on MOUD, implicit and explicit age-based biases still appear to be shaping MOUD prescribing practices [42]. Specific prescriber education and messaging that affirm MOUD as effective and clinically indicated for TA adults should be implemented to address the biases and misconceptions contributing to suboptimal treatment in this population [43].

### Indirect, life-stage influences of age on MOUD selection

Rather than relying primarily on chronological age, prescribers more commonly described MOUD type selection as being shaped by life-stage characteristics that can occur at any age but are more prevalent during TA adulthood, including neurobiological developmental factors, social context, and MOUD treatment feasibility. For example, when discussing developmental readiness, prescribers frequently described TA adults as appearing “uncommitted” to MOUD treatment. This perception aligns with prior research showing younger patients often have lower intrinsic motivation for recovery compared to adults ≥ 26 [44, 45]. However, this framing is problematic and potentially dangerous. OUD is a chronic and relapsing disease that is often compounded by trauma and developmental factors, including ongoing neurodevelopment during early adulthood that may influence impulse control and treatment engagement. [46–49]. Characterizing patients as “uncommitted” reflects a misunderstanding of the natural illness course of OUD and the developmental context of TA adulthood, risking the withholding of lifesaving MOUD during a particularly vulnerable period. In response to this reported “ambivalence”, MOUD treatment for TA adults should be adapted to meet patients where they are by reducing treatment barriers and supporting gradual progress rather than requiring full commitment as a condition of care.

Prescribers also viewed that social context, particularly peer influence and stigma, can shape TA adults’ engagement with MOUD. Since TA adults’ decisions about treatment are strongly influenced by social context, they may be particularly affected by peers’ attitudes toward MOUD and the type of MOUD they prefer. These prescriber observations align with evidence showing that TA adults are more likely to be influenced by social circles [50]. Peer attitudes may alter what MOUD type patients are willing to receive, if any, and lead prescribers to alter their MOUD prescribing accordingly to patient preference. Ultimately, prescribers described TA adults’ attitudes toward MOUD as a balance between persistent stigma and increasing cultural openness, with peer influence acting as a key determinant in either direction. Given the heightened influence of peers during TA adulthood, incorporating peer-based interventions may be particularly effective in facilitating MOUD engagement for this population, and efforts to incorporate these types of interventions should be implemented whenever possible.

Our findings additionally contextualize how low socioeconomic status and structural barriers contribute to treatment discontinuation among TA adults [51, 52]. Prescribers noted that addiction clinics often have stricter treatment requirements and lower discharge thresholds than other specialties, leaving little flexibility to accommodate the life challenges and responsibilities that disproportionately affect those with unmet social needs [53]. Although these structural barriers affect many patients, they may affect TA adults particularly hard. TA adults are more likely to have lower socioeconomic status and limited access to reliable transportation, increasing their likelihood of missing medical appointments [54]. Because of this, addiction treatment programs serving TA adults should adopt harm reduction frameworks that prioritize sustained access and engagement over rigid rules, including reconsideration of low-threshold discharge practices that may disproportionately set this population up to fail.

Our findings also indicate that family engagement is a factor that may play a role in shaping prescribers’ MOUD type selection for TA adults. Prescribers described that MOUD type and tapering plans were at times modified in response to familial dynamics, particularly when TA adults were financially or residentially dependent on relatives. When this occurred, prescribers viewed family opposition to buprenorphine and methadone as influencing patients’ expressed preferences for MOUD and, in response, influencing prescribers’ clinical decisions. Although these findings reflect prescribers’ perceptions rather than patient-reported experiences, they suggest that family involvement plays a meaningful role in shaping clinical decision-making. Because family influence is often closely tied to housing and economic dependence during TA adulthood, it may function as an indirect but consequential factor in MOUD selection for some TA adults. This dynamic may be stronger during TA adulthood, when reliance on family support is more common than in later adulthood [55, 56]. These findings suggest that family plays an important role in TA adult MOUD treatment. Interventions aimed at educating families and creating a supportive environment, particularly for agonist treatment with buprenorphine and methadone, may promote greater MOUD initiation and reduce disparities in agonist receipt for this age group [57, 58]. While our findings focus on TA adults, the role of family influence highlighted here shows the need for future research on family dynamics in MOUD decision-making among adolescents under 18, as caregiver involvement and limited autonomy likely play an even bigger role in MOUD treatment type and receipt for this population.

In contrast to peer and family influences on MOUD attitudes, some prescribers described direct patient stigma toward long-term buprenorphine and methadone treatment due to personal fears of dependency and lifelong medication use. These concerns align with previous work describing stigma against long-term MOUD maintenance treatment and previous evidence that has shown that this stigma may be stronger among young people [39, 45]. This stigma towards long-term MOUD maintenance treatment is significant, as hesitancy toward buprenorphine and methadone may lead clinicians to prescribe other less effective treatment alternatives. Clinicians often seek to align treatment with both perceived and explicitly expressed patient preferences, and as described in the results, some prescribers reported prescribing naltrexone over agonist MOUD therapies when TA adults expressed strong resistance to buprenorphine or methadone. If TA adults are more likely to express long-term buprenorphine and methadone treatment stigma than older adults, these patient-level preferences may represent an additional mechanism contributing to the higher relative receipt of naltrexone in this population. Future studies should evaluate whether age-related differences in MOUD prescribing continue even after controlling for patient preferences, which would help distinguish preference-driven decisions from prescriber or system-level contributions to age-based disparities in MOUD type received.

### Limitations

This study has several limitations. While the study findings may be generalizable to prescribers with substantial experience, they may differ from the perspectives of prescribers with less experience prescribing MOUD to TA adults. This study also excludes those who rarely or never prescribe to this age group, who may hold insights about the highest barriers to treatment initiation. However, the sampling approach was intentional to capture nuanced, practice-based insights from providers with repeated experience rather than hypothetical perspectives, as a broader sample could have introduced greater variability that may have diluted these findings. Notably, the 104 prescribers who met the ≥ 5 TA adult patient threshold in our study's sampling population accounted for approximately 33% of all buprenorphine and naltrexone prescriptions to TA adults in the New York State Medicaid dataset. This highlights that our sample represents prescribers treating a substantial proportion of TA adults accessing MOUD and has a central role in MOUD treatment for this population.

Although prescribers were prompted to recall on experiences with 18—25 year olds, some responses may have reflected experiences with younger or older patients not precisely in the 18–25 age range. This variability may have introduced some ambiguity in the interpretation of age-specific practices. Additionally, the study sample had adequate racial diversity; however, the sample underrepresented male prescribers and had limited representation from New York City, reducing generalizability to hyperdense urban or more diverse populations.

Although the reliance on Medicaid data allowed us to select our study sample of outpatient providers, the sample excludes prescribers who do not accept Medicaid and those at OTPs, potentially skewing the sample toward providers serving patients with greater structural barriers while limiting discussion of direct methadone prescribing practices. Accordingly, prescriber perspectives on methadone in this study largely reflect referral decision-making and perceptions rather than direct methadone prescribing experience. However, methadone represents a small minority of MOUD treatment among TA adults, suggesting that our sample captured prescriber perspectives on the MOUD treatments most used in this population [59].

Also, the findings related to TA adults’ perceptions and attitudes toward MOUD are based on prescribers’ reports rather than TA adults themselves. These findings reflect how prescribers remember and interpret patient attitudes and may be influenced by recall bias or clinicians’ assumptions. While these perceptions are important because they shape prescribing decisions, they may not represent TA adults’ actual experiences or preferences. Future research should be conducted with TA adults to see if the attitudes described by prescribers align with patients’ own perspectives related to MOUD treatment and the themes discussed in this paper. Finally, selection and response biases are likely, as participants may have had greater interest in research or more favorable attitudes toward MOUD than non-participants.

## Conclusion

This study demonstrates how age intersects with structural and social factors to shape MOUD prescribing for TA adults. While explicit age-related hesitancy toward prescribing MOUD was present among some prescribers, it was not the prevailing attitude expressed. Instead, prescribers more commonly described how prescribing decisions were influenced by age-related factors, including patients’ negative perceptions of MOUD, developmental stage, social influences, and living situations. These mechanisms may help explain the documented disparities in MOUD type prescribed to TA adults. Consistent with the themes reported by prescribers, interventions to reduce MOUD-type prescribing disparities should include prescriber education emphasizing buprenorphine and methadone as evidence-based MOUD for TA adults, efforts to reduce misconceptions about MOUD among TA adult patients and their families, and peer-focused approaches that leverage the strong influence of social networks during this life stage. Additionally, developmentally informed clinic policies prioritizing harm reduction by encouraging flexibility in care delivery and reducing rigid attendance requirements may better support MOUD access and retention for this population. By recognizing and responding to the age-specific challenges described by prescribers, care can be delivered in a way that is evidence-based and responsive to the needs of TA adults.

## Data Availability

The Medicaid claims data used in this study were accessed under a data use agreement and are not publicly available. The qualitative interview dataset contains identifiable information and cannot be shared publicly due to confidentiality protections. De-identified excerpts relevant to the study findings are included within the article. Additional information may be made available from the corresponding author upon reasonable request and with appropriate institutional and data use approvals.

## Appendix A

Our objective for this sample was to identify prescribers with extensive and recent experience treating TA adults with MOUD, namely buprenorphine and naltrexone. Thus, we first needed to identify prescribers who prescribed buprenorphine and naltrexone and then determine those with the highest MOUD prescribing volume for this age group. To do so, we used Medicaid administrative data from the 2022 calendar year to identify these medical prescribers. TA adults in the Medicaid dataset were identified as individuals aged 18 to 25 as of January 1, 2022. To create the sample, we extracted patient pharmacy claims from the 2022 Medicaid dataset and filtered for prescription fills associated with Buprenorphine and Naltrexone, identified by their National Drug Codes (NDCs). Using the most recent service date, we attempted to capture active prescriptions and then proceeded to exclude claims lacking valid or complete prescriber IDs. Prescriber IDs are linked to medical professionals in the State of New York who are licensed to prescribe medication and include nurse practitioners (NPs), physician assistants (PAs), doctors of osteopathy (DOs), and medical doctors (MDs). Within this Medicaid dataset, a total of 65,878 Medicaid patients had prescriptions for Buprenorphine or Naltrexone in 2022, with 96% (63,325 individuals) having valid information about their prescribers. Among these individuals, 2,296 met the criteria for TA adults, with their prescriptions linked to 1,088 unique prescriber IDs.

To capture prescribers with substantial experience treating TA adults, we defined these prescribers with substantial experience as those who prescribed either buprenorphine or naltrexone to at least five TA adults in the Medicaid dataset within the 2022 year. We chose this n ≥ 5 threshold to ensure that prescribers had sufficient clinical exposure to speak knowledgeably about the unique challenges and considerations involved in treating this population. At the same time, this threshold balanced expertise with variability, allowing us to maintain a sample broad enough to capture diverse perspectives and practice settings. A higher cutoff could have provided the sample with more specialized insights but would also have reduced the generalizability by excluding prescribers who prescribe MOUD to TA adults at lower but still meaningful levels. On the other hand, a lower threshold could have included more diversity of prescribers but would also have included those with little or incidental experience, potentially diluting their ability to speak in depth about prescribing to this population. Applying this n ≥ 5 criterion to the list of 1,088 prescriber IDs associated with TA adults, we identified a subset of 104 prescribers with substantial experience prescribing to TA adults. This method was used to create the final list of prescribers who were ultimately contacted for recruitment.

## Abbreviations

Agonist MOUD: Methadone and Buprenorphine
DO: Doctor of Osteopathy
LAI: Long-Acting Injectable
MD: Medical Doctor
MOUD: Medications for Opioid Use Disorder
NP: Nurse Practitioner
OUD: Opioid Use Disorder
OTP: Opioid Treatment Program
PA: Physician Assistant
TA: Transition-Age
SUD: Substance Use Disorder

## References

[1] Paul N, Kennedy AJ, Taubenberger S, Chang JC, Hacker K. Provider perceptions of medication for opioid used disorder (MOUD): A qualitative study in communities with high opioid overdose death rates. Subst Abuse [Internet]. 2022 [cited 2025 Jul 22];43(1):742–8. Available from: https://www.ncbi.nlm.nih.gov/pmc/articles/PMC10960355/10.1080/08897077.2021.2007518 PMC10960355 35100094

[2] Rosenblatt RA, Andrilla CHA, Catlin M, Larson EH. Geographic and Specialty Distribution of US Physicians Trained to Treat Opioid Use Disorder. Ann Fam Med [Internet]. 2015 Jan [cited 2025 Jul 22];13(1):23–6. Available from: https://www.ncbi.nlm.nih.gov/pmc/articles/PMC4291261/10.1370/afm.1735 PMC4291261 25583888

[3] Franz B, Dhanani LY, Hall OT, Brook DL, Simon JE, Miller WC. Differences in buprenorphine prescribing readiness among primary care professionals with and without X-waiver training in the US. Harm Reduct J. 2023;20(1):180. 10.1186/s12954-023-00918-3. 38129903 10.1186/s12954-023-00918-3 PMC10740221

[4] Mauro PM, Gutkind S, Annunziato EM, Samples H. Use of medication for opioid use disorder among US adolescents and adults with need for opioid treatment, 2019. JAMA Netw Open. 2022;5(3):e223821. 10.1001/jamanetworkopen.2022.3821. 35319762 10.1001/jamanetworkopen.2022.3821 PMC8943638

[5] Becker SJ, Scott K, Helseth SA, Danko KJ, Balk EM, Saldanha IJ, et al. Effectiveness of medication for opioid use disorders in transition-age youth: A systematic review. J Subst Abuse Treat [Internet]. 2022 Jan 1 [cited 2025 Jan 2];132:108494. Available from: https://www.sciencedirect.com/science/article/pii/S0740547221002208 10.1016/j.jsat.2021.108494 PMC8628023 34098208

[6] Sordo L, Barrio G, Bravo MJ, Indave BI, Degenhardt L, Wiessing L, et al. Mortality risk during and after opioid substitution treatment: systematic review and meta-analysis of cohort studies. BMJ. 2017;26(357):j1550. 10.1136/bmj.j1550 PMC5421454 28446428

[7] Welsh JW, Dennis ML, Funk R, Mataczynski MJ, Godley MD. Trends and age-related disparities in opioid use disorder treatment admissions for adolescents and young adults. J Subst Abuse Treat [Internet]. 2022 Jan [cited 2025 Jul 23];132:108584. Available from: https://www.ncbi.nlm.nih.gov/pmc/articles/PMC8671231/10.1016/j.jsat.2021.108584 PMC8671231 34391589

[8] Chadi N, Bagley SM, Hadland SE. Addressing Adolescents’ and Young Adults’ Substance Use Disorders. Med Clin North Am [Internet]. 2018 Jul 1 [cited 2024 Dec 23];102(4):603–20. Available from: https://www.sciencedirect.com/science/article/pii/S0025712518300191 10.1016/j.mcna.2018.02.015 29933818

[9] Stone AL, Becker LG, Huber AM, Catalano RF. Review of risk and protective factors of substance use and problem use in emerging adulthood. Addict Behav. 2012;37(7):747–75. 22445418 10.1016/j.addbeh.2012.02.014

[10] Gomes T, Ledlie S, Tadrous M, Mamdani M, Paterson JM, Juurlink DN. Trends in Opioid Toxicity-Related Deaths in the US Before and After the Start of the COVID-19 Pandemic, 2011–2021. JAMA Netw Open. 2023;6(7):e2322303. 37418260 10.1001/jamanetworkopen.2023.22303 PMC10329206

[11] Hudgins JD, Porter JJ, Monuteaux MC, Bourgeois FT. Prescription opioid use and misuse among adolescents and young adults in the United States: a national survey study. PLoS Med. 2019;16(11):e1002922. 31689290 10.1371/journal.pmed.1002922 PMC6830740

[12] Xu D, Li J, Yan H. The rising tide of opioid use disorder: analyzing 30 years of epidemiological trends and socioeconomic factors in the U.S. Naunyn Schmiedebergs Arch Pharmacol. 2025. 10.1007/s00210-025-04813-5. 41258062 10.1007/s00210-025-04813-5

[13] Volkow ND, Jones EB, Einstein EB, Wargo EM. Prevention and treatment of opioid misuse and addiction: a review. JAMA Psychiat. 2019;76(2):208–16. 10.1001/jamapsychiatry.2018.3126. 10.1001/jamapsychiatry.2018.3126 30516809

[14] Weiner SG, El Ibrahimi S, Hendricks MA, Hallvik SE, Hildebran C, Fischer MA, et al. Factors associated with opioid overdose after an initial opioid prescription. JAMA Netw Open. 2022;5(1):e2145691. 10.1001/jamanetworkopen.2021.45691. 35089351 10.1001/jamanetworkopen.2021.45691 PMC8800077

[15] Marsch LA, Bickel WK, Badger GJ, Stothart ME, Quesnel KJ, Stanger C, et al. Comparison of pharmacological treatments for opioid-dependent adolescents: a randomized controlled trial. Arch Gen Psychiatry. 2005;62(10):1157–64. 16203961 10.1001/archpsyc.62.10.1157

[16] Hadland SE, Wharam JF, Schuster MA, Zhang F, Samet JH, Larochelle MR. Trends in Receipt of Buprenorphine and Naltrexone for Opioid Use Disorder Among Adolescents and Young Adults, 2001–2014. JAMA Pediatr. 2017;171(8):747–55. 28628701 10.1001/jamapediatrics.2017.0745 PMC5649381

[17] Burns M, Tang L, Chang CCH, Kim JY, Ahrens K, Allen L, et al. Duration of medication treatment for opioid-use disorder and risk of overdose among Medicaid enrollees in 11 states: a retrospective cohort study. Addiction. 2022;117(12):3079–88. 35652681 10.1111/add.15959 PMC10683938

[18] Treitler P, Samples H, Hermida R, Miles J, Stone EM, Crystal S. Pharmacotherapy and overdose risk following new opioid use disorder diagnosis among Medicaid beneficiaries. Am J Drug Alcohol Abuse. 2025;23:1–11. 10.1080/00952990.2025.2557917 PMC12743245 41276914

[19] Santo T Jr, Clark B, Hickman M, Grebely J, Campbell G, Sordo L, et al. Association of opioid agonist treatment with all-cause mortality and specific causes of death among people with opioid dependence: a systematic review and meta-analysis. JAMA Psychiat. 2021;78(9):979–93. 10.1001/jamapsychiatry.2021.0976. 10.1001/jamapsychiatry.2021.0976 PMC8173472 34076676

[20] Dowell D, Brown S, Gyawali S, Hoenig J, Ko J, Mikosz C, et al. Treatment for Opioid Use Disorder: Population Estimates — United States, 2022. Morb Mortal Wkly Rep [Internet]. 2024 Jun 27 [cited 2026 Feb 2];73(25):567–74. Available from: https://pmc.ncbi.nlm.nih.gov/articles/PMC11254342/10.15585/mmwr.mm7325a1 PMC11254342 38935567

[21] Bagley SM, Larochelle MR, Xuan Z, Wang N, Patel A, Bernson D, et al. Characteristics of and Receipt of Medication Treatment among Young Adults Who Experience a Nonfatal Opioid-Related Overdose. Ann Emerg Med [Internet]. 2020 Jan [cited 2024 Dec 24];75(1):29–38. Available from: https://www.ncbi.nlm.nih.gov/pmc/articles/PMC7953238/10.1016/j.annemergmed.2019.07.030 PMC7953238 31591014

[22] Babu KM, Brent J, Juurlink DN. Prevention of Opioid Overdose. N Engl J Med [Internet]. 2019 Jun 6 [cited 2026 Feb 2];380(23):2246–55. Available from: https://www.nejm.org/doi/full/10.1056/NEJMra1807054 10.1056/NEJMra1807054 31167053

[23] Nosyk B, Min JE, Homayra F, Kurz M, Guerra-Alejos BC, Yan R, et al. Buprenorphine/Naloxone vs Methadone for the treatment of opioid use disorder. JAMA. 2024;332(21):1822–31. 10.1001/jama.2024.16954. 39418046 10.1001/jama.2024.16954 PMC11581542

[24] Frank CJ, Morrison L. Harm Reduction for Patients With Substance Use Disorders. Am Fam Physician [Internet]. 2022 Jan [cited 2026 Feb 5];105(1):90–2. Available from: https://www.aafp.org/pubs/afp/issues/2022/0100/p90.html 35029937

[25] Krawczyk N, Rivera BD, Jent V, Keyes KM, Jones CM, Cerdá M. Has the treatment gap for opioid use disorder narrowed in the U.S.?: A yearly assessment from 2010 to 2019”. Int J Drug Policy [Internet]. 2022 Dec [cited 2026 Feb 6];110:103786. Available from: https://pmc.ncbi.nlm.nih.gov/articles/PMC10976290/10.1016/j.drugpo.2022.103786 PMC10976290 35934583

[26] Palinkas LA, Horwitz SM, Green CA, Wisdom JP, Duan N, Hoagwood K. Purposeful sampling for qualitative data collection and analysis in mixed method implementation research. Adm Policy Ment Health. 2015;42(5):533–44. 10.1007/s10488-013-0528-y. 24193818 10.1007/s10488-013-0528-y PMC4012002

[27] Aleksanyan J, Choi S, Lincourt P, Burke C, Ramsey KS, Hussain S, et al. Lost in transition: a protocol for a retrospective, longitudinal cohort study for addressing challenges in opioid treatment for transition-age adults. PLoS ONE. 2024;19(8):e0297567. 39141672 10.1371/journal.pone.0297567 PMC11324150

[28] Saunders B, Sim J, Kingstone T, Baker S, Waterfield J, Bartlam B, et al. Saturation in qualitative research: exploring its conceptualization and operationalization. Qual Quant [Internet]. 2018 [cited 2025 Apr 2];52(4):1893–907. Available from: https://www.ncbi.nlm.nih.gov/pmc/articles/PMC5993836/10.1007/s11135-017-0574-8 PMC5993836 29937585

[29] Kallio H, Pietilä AM, Johnson M, Kangasniemi M. Systematic methodological review: developing a framework for a qualitative semi-structured interview guide. J Adv Nurs [Internet]. 2016 Dec 1 [cited 2025 Apr 14];72(12):2954–65. Available from: https://onlinelibrary.wiley.com/doi/10.1111/jan.13031 10.1111/jan.13031 27221824

[30] Paul N, Kennedy AJ, Taubenberger S, Chang JC, Hacker K. Provider perceptions of medication for opioid used disorder (MOUD): A qualitative study in communities with high opioid overdose death rates. Subst Abuse [Internet]. 2022 [cited 2025 Feb 17];43(1):742–8. Available from: https://www.ncbi.nlm.nih.gov/pmc/articles/PMC10960355/10.1080/08897077.2021.2007518 PMC10960355 35100094

[31] Buchholz C, Bell LA, Adatia S, Bagley SM, Wilens TE, Nurani A, et al. Medications for Opioid Use Disorder for Youth: Patient, Caregiver, and Clinician Perspectives. J Adolesc Health [Internet]. 2024 Feb 1 [cited 2025 Feb 25];74(2):320–6. Available from: https://www.sciencedirect.com/science/article/pii/S1054139X23004603 10.1016/j.jadohealth.2023.08.047 PMC10842045 37815763

[32] Nowell LS, Norris JM, White DE, Moules NJ. Thematic Analysis: Striving to Meet the Trustworthiness Criteria. Int J Qual Methods [Internet]. 2017 Dec 1 [cited 2025 Feb 25];16(1):1609406917733847. Available from: 10.1177/1609406917733847

[33] O’Connor C, Joffe H. Intercoder reliability in qualitative research: debates and practical guidelines. Int J Qual Methods. 2020;19:1609406919899220. 10.1177/1609406919899220.

[34] New York Executive Law § 481(7) [Internet]. 2014 [cited 2025 Feb 26]. Available from: https://www.nysenate.gov/legislation/laws/EXC/481

[35] Hadland SE, Aalsma MC, Akgül S, Alinsky RH, Bruner A, Chadi N, et al. Medication for Adolescents and Young Adults with Opioid Use Disorder. J Adolesc Health Off Publ Soc Adolesc Med [Internet]. 2021 Mar [cited 2024 Dec 24];68(3):632–6. Available from: https://www.ncbi.nlm.nih.gov/pmc/articles/PMC7902443/10.1016/j.jadohealth.2020.12.129 PMC7902443 33485735

[36] Carl A, Pasman E, Broman MJ, Lister JJ, Agius E, Resko SM. Experiences of healthcare and substance use treatment provider-based stigma among patients receiving methadone. Drug Alcohol Depend Rep [Internet]. 2023 Feb 7 [cited 2025 Apr 1];6:100138. Available from: https://www.ncbi.nlm.nih.gov/pmc/articles/PMC10040326/10.1016/j.dadr.2023.100138 PMC10040326 36994374

[37] Sabin JA, Marini M, Nosek BA. Implicit and Explicit Anti-Fat Bias among a Large Sample of Medical Doctors by BMI, Race/Ethnicity and Gender. PLoS ONE [Internet]. 2012 Nov 7 [cited 2025 Apr 1];7(11):e48448. Available from: https://www.ncbi.nlm.nih.gov/pmc/articles/PMC3492331/10.1371/journal.pone.0048448 PMC3492331 23144885

[38] FitzGerald C, Hurst S. Implicit bias in healthcare professionals: a systematic review. BMC Med Ethics [Internet]. 2017 Mar 1 [cited 2025 Apr 1];18(1):19. Available from: 10.1186/s12910-017-0179-8 10.1186/s12910-017-0179-8 PMC5333436 28249596

[39] Dickson-Gomez J, Spector A, Weeks M, Galletly C, McDonald M, Green Montaque HD. “You’re not supposed to be on it forever”: medications to treat opioid use disorder (MOUD) related stigma among drug treatment providers and people who use opioids. Subst Abuse Res Treat. 2022;16:11782218221103859. 10.1177/11782218221103859 PMC9243471 35783464

[40] Madden EF. Intervention stigma: how medication-assisted treatment marginalizes patients and providers. Soc Sci Med. 2019;232:324–31. 31125801 10.1016/j.socscimed.2019.05.027

[41] Hadland SE, Kimmel SD, Yan S, Bettano AL, Lo-Ciganic WH, Bagley SM, et al. Buprenorphine Treatment Duration and Adherence Among Youth and Subsequent Health Outcomes. Pediatrics. 2025;156(6):e2025071147. 41248890 10.1542/peds.2025-071147 PMC12662019

[42] Federal Register :: Medications for the Treatment of Opioid Use Disorder [Internet]. [cited 2025 Feb 17]. Available from: https://www.federalregister.gov/documents/2024/02/02/2024-01693/medications-for-the-treatment-of-opioid-use-disorder

[43] Mazzarelli S, Blewer AL, Østbye T, Rhodes K, Plasencia G, Hart L, et al. Impact of implementing primary care-based medication for opioid use disorder on provider and staff perceptions. Fam Pract. 2024;41(6):1018–24. 39312395 10.1093/fampra/cmae044 PMC11638083

[44] Fishman M, Wenzel K, Gauthier P, Borodovsky J, Murray O, Subramaniam G, et al. Engagement, initiation, and retention in medication treatment for opioid use disorder among young adults: A narrative review of challenges and opportunities. J Subst Use Addict Treat [Internet]. 2024 Nov 1 [cited 2025 Feb 17];166:209352. Available from: https://www.sciencedirect.com/science/article/pii/S294987592400064X 10.1016/j.josat.2024.209352 PMC11392652 38494051

[45] Bagley SM, Schoenberger SF, dellaBitta V, Lunze K, Barron K, Hadland SE, et al. Ambivalence and stigma beliefs about medication treatment among young adults with opioid use disorder: a qualitative exploration of young adults’ perspectives. J Adolesc Health Off Publ Soc Adolesc Med [Internet]. 2023 Jan [cited 2025 Feb 17];72(1):105–10. Available from: https://www.ncbi.nlm.nih.gov/pmc/articles/PMC10077517/10.1016/j.jadohealth.2022.08.026 PMC10077517 36216678

[46] Bonnie RJ, Stroud C, Breiner H, Committee on Improving the Health S, Board on Children Y, Medicine I of, et al. Young Adults in the 21st Century. In: Investing in the Health and Well-Being of Young Adults [Internet]. National Academies Press (US); 2015 [cited 2026 Feb 4]. Available from: https://www.ncbi.nlm.nih.gov/books/NBK284782/25855847

[47] Moss HB, Ge S, Trager E, Saavedra M, Yau M, Ijeaku I, et al. Risk for Substance Use Disorders in young adulthood: Associations with developmental experiences of homelessness, foster care, and adverse childhood experiences. Compr Psychiatry [Internet]. 2020 Jul 1 [cited 2026 Feb 4];100:152175. Available from: https://www.sciencedirect.com/science/article/pii/S0010440X20300171 10.1016/j.comppsych.2020.152175 32345436

[48] Knežević M, Marinković K. Neurodynamic correlates of response inhibition from emerging to mid adulthood. Cogn Dev. 2017;43:106–18. 29081593 10.1016/j.cogdev.2017.03.002 PMC5658138

[49] Domínguez-Salas S, Díaz-Batanero C, Lozano-Rojas OM, Verdejo-García A. Impact of general cognition and executive function deficits on addiction treatment outcomes: systematic review and discussion of neurocognitive pathways. Neurosci Biobehav Rev. 2016;71:772–801. 27793597 10.1016/j.neubiorev.2016.09.030

[50] Castrellon JJ, Zald DH, Samanez-Larkin GR, Seaman KL. Adult age-related differences in susceptibility to social conformity pressures in self-control over daily desires. Psychol Aging. 2024;39(1):102–12. 38059928 10.1037/pag0000790 PMC10922454

[51] Bureau of Labor Statistics [Internet]. [cited 2025 Feb 17]. A-10. Unemployment rates by age, sex, and marital status, seasonally adjusted. Available from: https://www.bls.gov/web/empsit/cpseea10.htm

[52] Grubb LK. Personal and Socioeconomic Determinants in Medication-assisted Treatment of Opioid Use Disorder in Adolescents and Young Adults. Clin Ther [Internet]. 2019 Sep 1 [cited 2025 Feb 17];41(9):1669–80. Available from: https://www.sciencedirect.com/science/article/pii/S0149291819303923 10.1016/j.clinthera.2019.07.019 31462387

[53] White WL, Scott CK, Dennis ML, Boyle MG. It’s Time to Stop Kicking People Out of Addiction Treatment. Couns Deerfield Beach Fla [Internet]. 2005 Apr [cited 2025 Feb 17];6(2):12–25. Available from: https://www.ncbi.nlm.nih.gov/pmc/articles/PMC6338434/PMC6338434 30662372

[54] Fiori KP, Heller CG, Rehm CD, Parsons A, Flattau A, Braganza S, et al. Unmet Social Needs and No-Show Visits in Primary Care in a US Northeastern Urban Health System, 2018–2019. Am J Public Health [Internet]. 2020 Jul [cited 2025 Apr 1];110(Suppl 2):S242–50. Available from: https://www.ncbi.nlm.nih.gov/pmc/articles/PMC7362703/10.2105/AJPH.2020.305717 PMC7362703 32663075

[55] Hogue A, Becker SJ, Wenzel K, Henderson CE, Bobek M, Levy S, et al. Family Involvement in Treatment and Recovery for Substance Use Disorders among Transition-Age Youth: Research Bedrocks and Opportunities. J Subst Abuse Treat [Internet]. 2021 Oct [cited 2025 Aug 14];129:108402. Available from: https://www.ncbi.nlm.nih.gov/pmc/articles/PMC8380649/10.1016/j.jsat.2021.108402 PMC8380649 34080559

[56] Aragão RM Kim Parker, Juliana Menasce Horowitz and Carolina. Parents, Young Adult Children and the Transition to Adulthood [Internet]. Pew Research Center. 2024 [cited 2025 Aug 14]. Available from: https://www.pewresearch.org/social-trends/2024/01/25/parents-young-adult-children-and-the-transition-to-adulthood/

[57] Porter NP, Dunnsue S, Hammond C, MacLean A, Bobek M, Watkins M, et al. “I need as much support as I can get”: a qualitative study of young adult perspectives on family involvement in treatment for opioid use disorder. J Subst Use Addict Treat. 2024;167:209512. 39265914 10.1016/j.josat.2024.209512 PMC11527569

[58] Bagley SM, Schoenberger SF, dellaBitta V, Lunze K, Barron K, Hadland SE, et al. An Exploration of Young Adults With Opioid Use Disorder and How Their Perceptions of Family Members’ Beliefs Affects Medication Treatment. J Addict Med [Internet]. 2022 [cited 2025 Feb 17];16(6):689–94. Available from: https://www.ncbi.nlm.nih.gov/pmc/articles/PMC9653106/10.1097/ADM.0000000000001001 PMC9653106 35749777

[59] Hadland SE, Bagley SM, Rodean J, Silverstein M, Levy S, Larochelle MR, et al. Receipt of timely addiction treatment and association of early medication treatment with retention in care among youths with opioid use disorder. JAMA Pediatr. 2018;172(11):1029–37. 10.1001/jamapediatrics.2018.2143. (**[cited 2026 Feb 5]**). 30208470 10.1001/jamapediatrics.2018.2143 PMC6218311

